# Measuring the Maturity of the Fast-Spiking Interneuron Transcriptional Program in Autism, Schizophrenia, and Bipolar Disorder

**DOI:** 10.1371/journal.pone.0041215

**Published:** 2012-08-24

**Authors:** Michael J. Gandal, Addie May Nesbitt, Richard M. McCurdy, Mark D. Alter

**Affiliations:** Center for Neurobiology and Behavior, Department of Psychiatry, University of Pennsylvania, Philadelphia, Pennsylvania, United States of America; Institute of Molecular and Cell Biology, Singapore

## Abstract

**Background:**

Emerging evidence suggests that fast-spiking (FS) interneurons are disrupted in multiple neuropsychiatric disorders including autism, schizophrenia, and bipolar disorder. FS cells, which are the primary source of synaptic inhibition, are critical for temporally organizing brain activity, regulating brain maturation, and modulating critical developmental periods in multiple cortical systems. Reduced expression of parvalbumin, a marker of mature FS cells, has been reported in individuals with schizophrenia and bipolar disorder and in mouse models of schizophrenia and autism. Although these results suggest that FS cells may be immature in neuropsychiatric disease, this possibility had not previously been formally assessed.

**Methods:**

This study used time-course global expression data from developing FS cells to create a maturation index that tracked with the developmental age of purified cortical FS cells. The FS cell maturation index was then applied to global gene expression data from human cortex to estimate the maturity of the FS cell developmental program in the context of various disease states. Specificity of the index for FS cells was supported by a highly significant correlation of maturation index measurements with parvalbumin expression levels that withstood correction for multiple covariates.

**Conclusions:**

Results suggest the FS cell developmental gene expression program is immature in autism, schizophrenia, and bipolar disorder. More broadly, the current study indicates that cell-type specific maturation indices can be used to measure the maturity of developmental programs even in data from mixed cell types such as those found in brain homogenates.

## Introduction

Several lines of evidence have suggested that fast-spiking interneurons (FS cells) are disrupted in neuropsychiatric disorders including autism, schizophrenia, and bipolar disorder [1], [2], [3], [4]. For instance, expression of parvalbumin, a marker of mature fast-spiking inhibitory neurons is decreased in individuals with schizophrenia and bipolar disorder and in several mouse models of schizophrenia and autism [1], [2], [3], [4]. Additionally, fast synaptic inhibition, which is largely mediated by fast-spiking interneurons, was significantly reduced in a mouse model of schizophrenia [5]. FS cells are well positioned to contribute to problems with neurodevelopment and brain function. These interneurons are the primary source of synaptic inhibition and help to temporally organize brain activity [6]. FS cells are also important for neuroplasticity and regulate critical periods of development in multiple cortical systems [7]. Previous studies have demonstrated pronounced phenotypic changes in multiple cellular properties as FS cells mature [8]. Thus, abnormal development of FS cells has the potential to impact brain function and to contribute to neuropsychiatric disorders.

Using cell-type selective fluorescent labeling followed by cell sorting, purification, and microarray analysis, developmental gene expression programs have been characterized in multiple cell types, including FS interneurons [8], [9], [10], [11]. As a general rule, significant changes in expression over time were observed for a majority of genes during development [8], [9], [10], [11]. In addition, expression level changes were primarily monotonic and highly stereotyped, indicating that most genes become increasingly upregulated or downregulated during development [8], [9], [10], [11]. As FS cells were hypothesized to be immature in autism, schizophrenia, and bipolar disorder, gene expression studies from these disorders could be assessed to see whether disease-related gene expression changes were consistent with an immature FS cell developmental program. To date, however, studies have not addressed this possibility and have instead focused on the identification of disease-related single gene expression differences without considering possible transcriptome-wide alterations in cell-type specific developmental programs that may more completely account for phenotypic differences in neuropsychiatric populations. This study investigated the hypothesis that the developmental gene expression program of FS cells is immature in the context of disorders in which research suggested there were immature FS cells.

## Methods

### Microarray data processing

Microarray data sets were obtained from the Gene Expression Omnibus (GEO - http://www.ncbi.nlm.nih.gov/geo/). In order that identical maturation indices could be applied to data sets derived from different microarray platforms, expression levels were referenced to universal gene symbols. In cases where there was more than one expression level for a given gene symbol, expression levels were averaged to give a single expression value per gene. When present calls were available (Affymetrix 3′ expression arrays), a weighted average was performed based on the percent present for each probe set. After obtaining single expression levels for each gene, genes were further restricted to those that had universal gene symbols and were assessed in all microarray platforms used in the current study (2,123 genes).

### Maturation indices

Data from time series gene expression profiling of developing FS cells, astrocytes, and corticospinal projection neurons were downloaded from GEO and used to create cell-type specific maturation indices as follows. For each cell type, genes were divided into groups based on whether they were upregulated or downregulated during development. To equally weight all genes, genes in an index were quantile normalized such that all genes had the same mean and variance. Because they would be used to assess FS cell index specificity, parvalbumin levels were excluded from the calculation of all indices. An index was calculated for a sample by taking the ratio of the average of genes that were upregulated in a cell type divided by the average of those that were downregulated.

### Statistics

Multiple regression analysis and student's t-tests were performed using Statview (SAS). Chi-square analysis was done using an online chi-square calculator (http://www.graphpad.com/quickcalcs/contingency1.cfm, Graphpad).

### Pathway Analysis

DAVID (david.abcc.ncifcrf.gov) Functional Annotation Bioinformatics Microarray Analysis [12], [13] was used to assess gene lists for enriched biological themes. A user supplied background list was uploaded which consisted of a list of single probesets for each gene used in the study (see above: 2,123 genes). Benjamini correction was used to account for multiple testing [12], [13].

## Results

### FS cell maturation index derived from developmental time-course expression data

**Figure 1:**
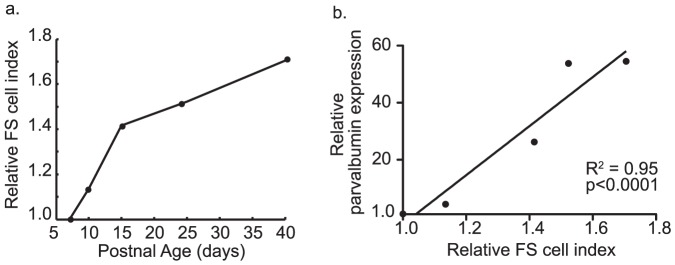
FS cell maturation index in developing FS cells. Panel (a) is a line plot of FS cell postnatal age (x-axis) versus the FS cell index (y-axis) in purified developing FS cells. Panel shows that the FS cell index increases with age. Panel (b) is a linear regression of parvalbumin expression levels versus the FS cell index. Index measurements and parvalbumin levels were highly correlated in developing FS cells (R^2^ = 0.95). Parvalbumin expression levels were excluded from the calculation of the FS cell index.

Time-course expression profiling data from parvalbumin-expressing, fast-spiking interneurons (FS cells) were publicly available from the Gene Expression Omnibus [8]. Expression levels for most genes changed monotonically (up or down) across development [8]. As such, the ratio of the average of developmentally upregulated over downregulated genes also increased monotonically with time (figure 1). The ratio of averaged upregulated (1023 genes) over averaged downregulated genes (1100 genes), therefore, could be used as a maturation index for the development of purified FS cells (figure 1). To make the index applicable to heterogeneous tissue with diverse cell types, gene expression levels were quantile normalized to give all genes equal weight. This assured that the index would vary only if a large number of genes changed expression levels in a non-random manner with respect to FS cell development. Further, as the levels of parvalbumin would be used to evaluate for specificity of the index, this gene was not included in index calculations. Despite the removal of parvalbumin, FS cell index levels were, nonetheless, highly correlated with parvalbumin expression levels (figure 1), indicating that the relationship between parvalbumin and the FS cell index might be used to assess for specificity of the index for parvalbumin-expressing FS cells in data from heterogeneous tissue.

### FS cell maturation index applied to data from developing prefrontal cortex

**Figure 2:**
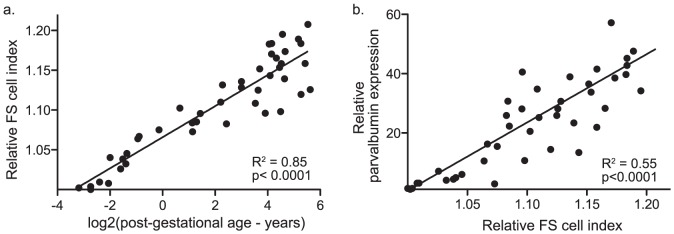
FS cell index applied to typically developing prefrontal cortex. Panel (a) is a linear regression of FS cell index measurements from developing human prefrontal cortex versus post-gestational age (log2 years). Panel shows that the FS cell index increases with age. Panel (b) is a linear regression of the FS cell index versus parvalbumin expression levels in developing prefrontal cortex. Panel shows that parvalbumin levels were closely related to FS cell index measurements even in data from heterogeneous tissue. Parvalbumin expression levels were excluded from the calculation of the FS cell index.

Next, to validate that the FS cell index could be used to track the maturation of FS cells in heterogeneous human tissue, the index was applied to microarray data collected during human prefrontal cortex (PFC) development [14]. Indeed, the FS cell index significantly correlated with subject age (Figure 2; R^2^ = 0.85, p<0.0001). An alternative interpretation, however, could be that similar expression changes in other cell types over development were responsible for changes in index measurements. To address this possibility, it was important to assess for specificity of the FS cell index. To do this, the full FS cell index was compared to a more restricted measure of FS cell maturity, parvalbumin expression levels. Parvalbumin is highly specific for FS cells in the cortex and increases dramatically across development [8], [15] (figure 1). If the FS index was tracking the maturity of the FS cell program, then the two measures would be correlated. Indeed, there was good agreement between the full FS index and parvalbumin levels (figure 2; R^2^ = 0.55, p<0.0001). A correlation with parvalbumin levels, however, did not rule out the possibility that the FS cell index was simply a non-specific marker of maturation and that an index of any maturing cell-type would be equally predictive of parvalbumin levels. To assess this possibility, two additional indices were created based on developmental gene expression profiling of astrocytes [10] and corticospinal projection neurons [11]. As would be expected, these indices also increased with subject age in prefrontal cortex, and, therefore, were also correlated with parvalbumin expression levels (table 1). However, when the 3 indices were entered into a multiple regression model with parvalbumin levels as the dependent variable, only the FS cell index remained significant (p<0.005) indicating that the FS index was the primary predictor of levels of the FS cell marker, parvalbumin (table 1).

**Table 1:** Multiple linear regression - Human prefrontal cortex development.

Dependent variable: parvalbumin expression levels			
Index	Coeff	Std Err	p-value
FS	6.33	0.89	<0.0001***
CSP	----	----	----
AST	----	----	----

Index	Coeff	Std Err	p-value
FS	----	----	----
CSP	1.74	0.34	<0.0001***
AST	----	----	----

Index	Coeff	Std Err	p-value
FS	----	----	----
CSP	----	----	----
AST	3.00	0.52	<0.0001***

Index	Coeff	Std Err	p-value
FS	5.27	1.61	0.002**
CSP	−0.55	0.81	0.500
AST	1.56	0.35	0.261

FS = FS cell index.

CSP = Corticospinal projection neuron index.

AST = Astrocyte index.

The first 3 tables show simple linear regression of 3 maturation indices (FS cell index, Corticospinal projection neuron index, and Astrocyte index) on parvalbumin expression levels in data from developing human prefrontal cortex. In all cases there was a highly significant relationship between index measurements and parvalbumin expression levels (p<0.0001). The fourth table shows results when all three indices were entered in the same model. Only the FS cell index remained significantly associated with parvalbumin expression levels (p<0.002).

### FS cell index is robust across microarray platforms and studies

**Figure 3:**
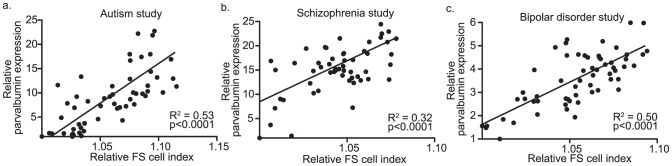
FS cell index versus parvalbumin expression levels in gene expression studies of neuropsychiatric disease. Panels (a–c) are linear regression plots of the FS cell index versus parvalbumin expression levels in 3 separate studies of global gene expression in autism(a), schizophrenia(b), and bipolar disorder(c). Panels show that the relationship between FS cell index measurements and parvalbumin levels is robust across studies and micoarray platforms. Parvalbumin expression levels were excluded from the calculation of the FS cell index.

Given that the FS cell maturation index was based on expression levels of 2000+ genes, we hypothesized that it would be robust to changes in microarray platforms and across studies. Indeed, results were similar when the FS cell index was applied to data from 3 studies of global gene expression in the neuropsychiatric disorders autism, schizophrenia, and bipolar disorder. The autism study included samples from temporal and frontal cortex and used an Illumina beadchip array, whereas, the schizophrenia and bipolar studies were done on dorsolateral prefrontal cortex with Affymetrix 3′ arrays. In all cases, there was good agreement between the full index and parvalbumin levels (figure 3). As in developing prefrontal cortex, multiple regression modeling indicated that only the FS cell index significantly explained variance in parvalbumin levels ( table S1). Moreover, in contrast to results from the prefrontal cortex development study, none of the other cell-type specific indices were positively correlated with parvalbumin expression levels (table S2). Thus, in non-developing brain, the FS cell index was the only index examined that positively correlated with parvalbumin levels, further supporting that the FS cell index was detecting global changes in the FS cell transcriptome within data from mixed cell types. As it was reported that other subsets of interneurons might be abnormal in neuropsychiatric disorders [16], multiple regression analysis was used to test whether the relationship between the FS cell index and parvalbmin levels could be accounted for by changes in the maturity of another population of interneurons, including those that express cholecystikinin (CCK), Calretinin (CALR), Somatostatin (SSTR1), and vasoactive intestinal peptide (VIP). If the FS cell index was solely driven by changes in another population of interneurons, then the relationship between the FS cell index and parvalbumin levels would no longer be significant after the marker for this other interneuron subset was added as a covariate to the multiple regression model. First, it was assessed what interneuron markers positively covaried with the FS cell index in the autism, schizophrenia, and bipolar studies. In all three studies, CCK and VIP positively correlated with the FS cell index (table S3). When these markers were entered as covariates into multiple regression models, the relationship between the FS cell index and parvalbumin remained significant in all cases (autism: p<0.0001; schizophrenia: p<0.02; bipolar: p<0.0002) (table S4). Thus, even after removing variance associated with other interneuron markers as covariates, a significant relationship between the FS cell index and parvalbumin levels remained in all cases.

### FS cell index is broad based

As equal weight was given to all genes within the FS cell index, it was expected that genes that contributed to variability in index measurements would be widely distributed across the transcriptome. A broadly distributed set of genes contributing to index variability would be consistent with variability in the FS cell program, whereas a limited number of genes would suggest targeted effects of disease on specific genes. If affected genes were spread throughout the transcriptome, then randomly selected sub-indices would be positively correlated with each other. To assess this, the FS cell index was divided into 20 randomly selected independent sub-indices that used the average of only 50 upregulated over 50 downregulated genes. Independent indices did not share any of the same genes. The values from these 20 independent indices were cross-correlated to give 180 unique comparisons. The actual probability of a positive correlation was compared to the probability of a positive correlation if the sub-indices were unrelated (probability = 0.5). There was a very high probability of a positive cross-correlation between randomly chosen independent indices, ranging from 82% (p<0.0001) in the schizophrenia study to 100% (p<0.0001) in the human PFC development study, indicating that expression levels contributing to index measurements were widely distributed. The autism study had a 95% (p<0.0001) probability of positive cross-correlation and random independent indices applied to data from the bipolar study were positively correlated 90% (p<0.0001) of the time.

### FS cell index is decreased in neuropsychiatric disorders

**Figure 4:**
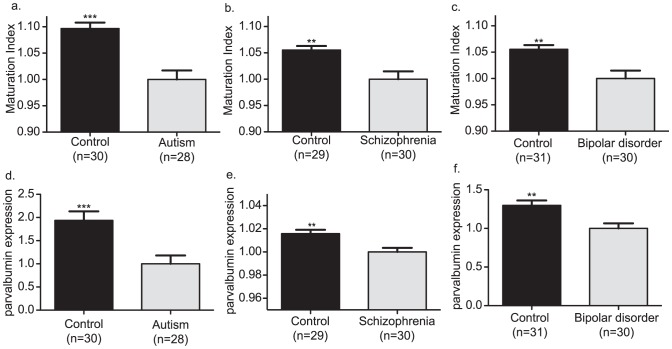
FS cell index in psychiatric disorders. Panels (a–c) are bar graphs of the FS cell index in the cortex of controls and individuals with disease. Panels show that the FS cell index was decreased in the cortex in autism, schizophrenia, and bipolar disorder. Panels (d–f) are bar graphs of parvalbumin levels in the cortex of controls and individuals with disease. Panels show that parvalbumin levels were decreased in the cortex in autism, schizophrenia, and bipolar disorder. Parvalbumin expression levels were excluded from the calculation of the FS cell index. ** p<0.01, ***p<0.001 n = number of subjects.

As previous work has suggested that FS cells are disrupted in multiple neuropsychiatric disorders, it was evaluated whether disease-related differences in index measures were present in the autism, schizophrenia, and bipolar disorder studies. The FS cell index was significantly decreased relative to controls in all 3 diseases (figure 4a–c), consistent with an immature FS cell program. Parvalbumin was also decreased in autism, schizophrenia, and bipolar disorder (figure 4d–f). All 20 randomly chosen sub-indices were decreased in the autism study (p<0.0001), 19/20 sub-indices were decreased in the bipolar study (p<0.0001), and in the schizophrenia study 17/20 (p<0.002) sub-indices were decreased, indicating that results were related to a large number of genes distributed throughout the transcriptome. A decrease in the FS cell index in autism, schizophrenia, and bipolar disorder suggested that gene expression changes in these disorders were in the opposite direction from that occurring during FS cell development. To confirm this was the case, a chi-square analysis was performed. All genes were used for the chi-square analysis and genes were split into groups of upregulated or downregulated genes with respect to disease and FS cell development. Using all genes was important because differences in a limited number of genes would not be able to affect chi-square results, and therefore, a significant result would mean genes contributing to the result were widely distributed. In agreement with results from the FS cell index, genes moved in the opposite direction as during FS cell development in all 3 diseases (p<0.0001) (table S5). Chi-square results remained highly significant when genes were restricted to only those that were significantly regulated in disease and FS cell development (p<0.0001) (table S5).

To understand how differences in FS cell development might impact a more traditional analysis of gene expression levels a number of predictions were examined. If the FS cell program was immature in multiple disease states and FS cell immaturity accounted for disease-related gene expression differences, then a significant overlap in gene expression changes between autism, bipolar disorder, and schizophrenia would be expected. Further, it would be predicted that overlapping genes would change expression in the same direction across disease states and that overlapping genes would change expression in an opposite direction from FS cell development. Finally, as gene expression levels of genes that were developmentally downregulated in FS cells might be obscured by expression levels of those genes in other cell types in heterogeneous brain tissue, it would be predicted that overlapping expression changes would be enriched for towards genes that were developmentally upregulated in FS cells. Indeed there was a highly significant overlap between gene expression changes in the 3 disease-related datasets. There were 137 genes that significantly changed expression levels in all 3 diseases compared to 49 genes that would be predicted by chance (p<0.0001). Of these, 136/137 genes (99.3%) changed expression in the same direction across all disease states (p<0.0001). Of the 136 genes that significantly changed expression in the same direction across disease states, 95 (69.9%) changed expression in the opposite direction from FS cell development (p<0.0001). Of the 95 genes that changed expression in the opposite direction from FS cell development, 60/95 (63.2%) genes were upregulated during FS cell development and only 35/95 (36.8%) genes were downregulated. The 95 genes (table s6) that changed expression levels in the same direction across disease and in an opposite direction from FS cell development were further examined with pathway analysis using DAVID [12], [13]. For genes that were upregulated during development and down in disease there were significant enrichments for energy related pathways including oxidative phosphorylation and ATP synthesis. Additionally there was enrichment for a mitochondrial pathway (table s7). Pathway enrichment withstood correction for multiple testing. Similar pathway enrichment was seen in subsets of genes used for the intersection including: 1) genes that were upregulated during FS development (1023 genes) or 2) downregulated in all disease states (85 genes) (table s7). With respect to genes that were downregulated during development and upregulated in disease there were no pathways that withstood correction for multiple testing.

## Discussion

Analyses suggested that gene expression changes in autism, schizophrenia, and bipolar disorder were consistent with an immature developmental gene expression program of parvalbumin-positive fast-spiking interneurons (FS cells). This conclusion was indicated by a robust relationship between a FS cell maturation index and parvalbumin expression levels across multiple studies and a decrease in both measures in association with autism, schizophrenia, and bipolar disorder.

The association of parvalbumin expression levels with the FS cell index was surprising because the FS cell index was broad-based and used equally weighted expression levels of all genes except for parvalbumin (see methods), whereas, the level of parvalbumin expression represented a very restricted developmentally regulated marker of FS cells. Though decreased parvalbumin levels had been reported in association with neuropsychiatric disorders [1], [2], [3], [4], it was unclear whether this decrease was gene specific or related to transciptome-wide changes in the FS cell developmental program. That a broad-based measure robustly correlated with the expression level of a single cell-type specific developmentally regulated gene is strongly suggestive of transriptome-wide co-regulation of a large number of genes and is consistent with a shift towards immaturity in the FS cell developmental program. The fact that genes were segregated in the FS index based solely on direction of gene expression change during FS cell maturation in purified cortical FS cells further suggested that the FS cell index reflected the maturity of the FS cell developmental program in cortex. This conclusion was also supported by the finding that the association between the FS cell index and parvalbumin remained significant after controlling for covariation with other cell type indices and with other interneuron specific markers. Finally, chi-square analyses provided an additional level of support for FS cell developmental program-associated gene expression changes in disease that were opposite in direction from changes that occurred during FS cell development.

An approach where averages of equally weighted genes are used to create a cell-type specific index is well suited to the detection of transcriptome-wide changes in the maturity of gene expression programs. Since all genes are weighted equally, only non-random gene expression differences with respect to program maturation can contribute to a change in the index, whereas, random changes will cancel out. Thus, an index measurement can only vary when a large percentage of genes move in a coordinated manner that is consistent in direction with expression level changes during development. Comparing variability in an index measure that relies on transcriptome-wide changes in gene expression levels to expression levels of an established cell-type specific marker, such as parvalbumin, can support specificity of the transcriptome-wide approach. Specificity can be further confirmed when the relationship between the index and marker withstands correction for covariation with other cell type specific indices and markers. The use of equally weighted gene expression levels to create the index also likely contributes to the robustness of the approach. In all cases, the FS cell index was closely related to parvalbumin levels regardless of array platform or species. Thus, the equal weighting and averaging of genes, it is hypothesized, makes it possible to capture the essence of redundant but noisy information about the state of developmental programs.

The robust correlation of the FS cell index with parvalbumin levels and their coordinated decrease in disease provides additional support for the hypothesis of FS cell program immaturity in disease that compliments previous experimental data consistent with this possibility. Numerous studies reported decreased parvalbumin levels in neuropsychiatric disease [1], [2], [3], [4]. While this could be interpreted to suggest decreased numbers of FS cells, evidence suggested FS cell immaturity. For instance, *in situ* hybridization in post-mortem brains of individuals with schizophrenia showed that parvalbumin-positive interneurons were still present in the same numbers but that levels of parvalbumin mRNA per cell were markedly reduced [2], [3]. Also, neurophysiological signatures of FS cells were immature in a mouse model of schizophrenia [5]. Thus, both the current data analyses and previous studies were consistent with the interpretation that the FS cell developmental gene expression program is immature in autism, schizophrenia, and bipolar disorder.

Current results also provide an interesting context to traditional studies of gene expression changes. For instance, a significant overlap of gene expression changes was found in autism, bipolar disorder, and schizophrenia with 137 genes significantly changing in all 3 diseases. Importantly, 99.3% of these overlapping genes moved in the same direction in all diseases and 69.9% of these moved in the opposite direction during FS cell development. Further, these genes were biased (63.2%) toward those that were upregulated during FS cell development. The unified direction of gene expression changes across disease states is suggestive of a similarly coordinated regulation of this set of genes across disease states. That this set of genes change in the opposite direction during FS cell development suggests abnormal regulation of FS cell maturation may be the causal mechanism behind these disease-related gene expression changes. Further supporting this idea was enrichment for developmentally upregulated genes that would be expected in a heterogeneous tissue such as brain where downregulated genes might be obscured by higher expression levels of those genes in other cell types. Suggesting, that abnormal FS maturation might be important to the interpretation of published studies, pathway analysis identified energy metabolism, oxidative phoshporylation, and mitochrondial function as overrepresented in the subset of genes that were downregulated in all diseases and upregulated during FS cell maturation. Mitochondrial dysfunction and abnormal measures of oxidative phosphorylation and energy metabolism have been widely reported in autism, bipolar disorder, and schizophrenia [17], [18], [19], [20], [21], [22], [23], [24]. FS cells have high metabolic demands and dramatically upregulated energy related genes during development (table s7). An interesting interpretation of these results might be that an underlying defect in FS cell maturation accounts for the widely reported disease-related abnormalities in oxidative phosphorylation and energy metabolism. It is also possible that primary defects in oxidative phosphorylation or energy metabolism genes might impair FS cell development.

Variability in transcriptional program maturity could have important implications for brain function. This is because the function of FS cells varies dramatically across development. FS cells have significant changes across development in firing frequency, firing type, firing rates, and resting membrane potential [8]. Thus, a change in the developmental age of FS cells would be expected to have functional consequences. A role for transcriptional immaturity in disease could also inform the study of risk factors. For instance, might prenatal viral infections associated with increased risk of neuropsychiatric disease [25], [26], [27] systematically interfere with the progression of developmental gene expression programs? Finally, the current study adds to the growing body of literature suggesting links between autism, schizophrenia, and bipolar disorder that were found to share genetic [28] and environmental risk factors [29], [30], [31], [32] as well as endophenotypes [33], [34], [35].

To conclude, the current study describes a novel method for assessing the maturity of the FS cell developmental program using gene expression data from mixed cell-types in brain homogenate. Use of this method suggested a new interpretation of changes in gene expression levels in neuropsychiatric disease to be at least partially related to an immature gene expression program in FS cells. Understanding mechanisms underlying impaired developmental program progression could improve understanding of disease pathogenesis.

## Supporting Information

Table S1 **Multiple regression models.** Multiple linear regression modeling was used to assess the relationship between parvalbumin levels and cell-type specific maturation indices. In all cases, the association between the FS cell index and parvalbumin expression levels remained highly significant (p<0.0001) after the Corticospinal projection neuron index (CSP) and Astrocyte index (AST) were included as covariates in the model.
(PDF)

Table S2 **Simple linear regression on FS cell index.** Simple linear regression was performed with parvalbumin expression levels as the dependent variable. In all cases the FS cell index was positively correlated with parvalbumin expression levels. Parvalbumin expression levels were not positively correlated with either the corticospinal or astrocyte indices in any of the disease-related gene expression studies. In the autism study there was a significant negative relationship between the corticospinal maturation index and parvalbumin expression levels. In the schizophrenia study there was a significant negative relationship between the astrocyte maturation index and parvalbumin expression levels. In the bipolar study there were significant negative correlations between parvalbumin expression levels and both the corticospinal and astrocyte maturation indices.
(PDF)

Table S3 **Simple linear regression models with interneuron markers.** Simple linear regression was performed with the FS cell index as the dependent variable. There was a significant positive correlation between parvalubmin expression levels and the FS cell index in all studies. The FS cell index was also positively correlated with CCK and VIP levels in all studies. Calretinin was negatively correlated with the FS cell index in the autism and bipolar studies.
(PDF)

Table S4 **Multiple regression models with interneuron markers.** Multiple linear regression modeling was performed with the FS cell index as the dependent variable. As CCK and VIP were also significantly correlated with the FS cell index in the autism, schizophrenia, and bipolar disorder studies, they were entered as covariates into multiple regression models. In all cases, the relationship between the FS cell index and parvalbumin levels remained significant.
(PDF)

Table S5 **Chi-square analysis.** For sub-tables 1–3, all genes were divided into 4 groups (up during FS cell development/up in disease; up during FS cell development/down in disease; down during FS cell development/up in disease; down during FS cell development/down in disease). Values were entered into an online chisquare 2×2 contingency table that used the Fisher's exact test for calculation of pvalues. In all cases, genes expression levels in the presence of disease changed in the opposite direction from that occurring during FS cell development (p<0.0001). For subtables 4–6, only genes that were significantly change in disease and during development were used. In all cases results remained highly significant (p<0.0001).
(PDF)

Table S6 **List of 95 genes that were significantly regulated in the same direction during disease and in an opposite direction during FS cell development.** Genes are separated into 2 groups based on the direction of regulation.
(PDF)

Table S7 **Top chart is pathway analysis using DAVID of the 60 genes that were upregulated during FS cell development and downregulated in autism, bipolar disorder, and schizophrenia.** Middle chart is pathway analysis using DAVID of 85 genes downregulated in autism, bipolar, and schizophrenia. Bottom chart is pathway analysis using DAVID of the 1023 genes that were upregulated in FS cells over development. Pathways enriched in all are starred.
(PDF)
