# Supplementary material for: Measuring the Maturity of the Fast-Spiking Interneuron Transcriptional Program in Autism, Schizophrenia, and Bipolar Disorder
Source: PLoS One. 2012 Aug 24;7(8):e41215. doi: 10.1371/journal.pone.0041215 (PMC3427326; doi:10.1371/journal.pone.0041215)
Supplement: Table S1 — Multiple regression models. Multiple linear regression modeling was used to assess the relationship between parvalbumin levels and cell-type specific maturation indices. In all cases, the association between the FS cell index and parvalbumin expression levels remained highly significant (p<0.0001) after the Corticospinal projection neuron index (CSP) and Astrocyte index (AST) were included as covariates in the model. (PDF) [file pone.0041215.s001.pdf]

Study: Autism cortex

Index	Coeff	Std Err	p-value
FS	23.40	5.05	p<0.0001***
CSP	-3.49	2.50	0.16
AST	1.41	3.38	0.679

Study: Schizophrenia dorsolateral prefrontal cortex

Index	Coeff	Std Err	p-value
FS	165.35	33.30	p<0.0001***
CSP	-12.22	9.36	0.197
AST	-6.44	21.47	0.766

Study: Bipolar dorsolateral prefrontal cortex

Index	Coeff	Std Err	p-value
FS	33.04	5.33	p<0.0001***
CSP	3.28	2.18	0.138
AST	-7.52	3.92	0.060

FS = FS cell index
CSP = Corticospinal projection neuron index
AST = Astrocyte index

Supplemental Table S1: Multiple regression models. Multiple linear regression modeling was used to assess the relationship between parvalbumin levels and cell-type specific maturation indices. In all cases, the association between the FS cell index and parvalbumin expression levels remained highly significant (p<0.0001) after the Corticospinal projection neuron index (CSP) and Astrocyte index (AST) were included as covariates in the model.
