# Supplementary material for: Measuring the Maturity of the Fast-Spiking Interneuron Transcriptional Program in Autism, Schizophrenia, and Bipolar Disorder
Source: PLoS One. 2012 Aug 24;7(8):e41215. doi: 10.1371/journal.pone.0041215 (PMC3427326; doi:10.1371/journal.pone.0041215)
Supplement: Table S2 — Simple linear regression on FS cell index. Simple linear regression was performed with parvalbumin expression levels as the dependent variable. In all cases the FS cell index was positively correlated with parvalbumin expression levels. Parvalbumin expression levels were not positively correlated with either the corticospinal or astrocyte indices in any of the disease-related gene expression studies. In the autism study there was a significant negative relationship between the corticospinal maturation index and parvalbumin expression levels. In the schizophrenia study there was a significant negative relationship between the astrocyte maturation index and parvalbumin expression levels. In the bipolar study there were significant negative correlations between parvalbumin expression levels and both the corticospinal and astrocyte maturation indices. (PDF) [file pone.0041215.s002.pdf]

Study: Autism cortex

Index	Coeff	Std Err	p-value
FS	26.985	4.376	<0.0001***
CSP	-6.534	2.022	0.0021**
AST	-1.809	3.239	0.5787

Study: Schizophrenia dorsolateral prefrontal cortex

Index	Coeff	Std Err	p-value
FS	168.553	32.251	<0.0001***
CSP	-14.120	8.777	0.1132
AST	-43.317	19.137	0.0274

Study: Bipolar dorsolateral prefrontal cortex

Index	Coeff	Std Err	p-value
FS	36.102	4.914	<0.0001***
CSP	-4.120	1.865	0.0310*
AST	-11.449	2.927	0.0002***

Supplemental Table 2: Simple linear regression on FS cell index. Simple linear regression was performed with parvalbumin expression levels as the dependent variable. In all cases the FS cell index was positively correlated with parvalbumin expression levels. Parvalbumin expression levels were not positively correlated with either the corticospinal or astrocyte indices in any of the disease-related gene expression studies. In the autism study there was a significant negative relationship between the corticospinal maturation index and parvalbumin expression levels. In the schizophrenia study there was a significant negative relationship between the astrocyte maturation index and parvalbumin expression levels. In the bipolar study there were significant negative correlations between parvalbumin expression levels and both the corticospinal and astrocyte maturation indices.
