# Supplementary material for: Measuring the Maturity of the Fast-Spiking Interneuron Transcriptional Program in Autism, Schizophrenia, and Bipolar Disorder
Source: PLoS One. 2012 Aug 24;7(8):e41215. doi: 10.1371/journal.pone.0041215 (PMC3427326; doi:10.1371/journal.pone.0041215)
Supplement: Table S3 — Simple linear regression models with interneuron markers. Simple linear regression was performed with the FS cell index as the dependent variable. There was a significant positive correlation between parvalubmin expression levels and the FS cell index in all studies. The FS cell index was also positively correlated with CCK and VIP levels in all studies. Calretinin was negatively correlated with the FS cell index in the autism and bipolar studies. (PDF) [file pone.0041215.s003.pdf]

Study: Autism cortex

Index	Coeff	Std Err	p-value
PVALB	0.015	0.002	<0.0001***
CALR	-0.080	0.012	<0.0001***
CCK	0.010	0.003	0.0003***
SSTR1	0.005	0.008	0.5503
VIP	0.008	0.002	<0.0001***

Study: Schizophrenia dorsolateral prefrontal cortex

Index	Coeff	Std Err	p-value
PVALB	0.002	0.0004	<0.0001***
CALR	0.001	0.006	0.9016
CCK	0.008	0.001	<0.0001***
SSTR1	0.004	0.002	0.0567
VIP	0.003	0.001	0.0011***

Study: Bipolar dorsolateral prefrontal cortex

Index	Coeff	Std Err	p-value
PVALB	0.014	0.002	<0.0001***
CALR	-0.039	0.009	0.0001***
CCK	0.016	0.003	<0.0001***
SSTR1	0.017	0.017	0.3193
VIP	0.047	0.010	<0.0001***

PVALB = parvalbumin
CALR = calretinin
CCK = cholecystokinin
SSTR1 = somatostatin
VIP = vasoactive intestinal peptide

Supplemental Table S3: Simple linear regression models with interneuron markers. Simple linear regression was performed with the FS cell index as the dependent variable. There was a significant positive correlation between parvalubmin expression levels and the FS cell index in all studies. The FS cell index was also positively correlated with CCK and VIP levels in all studies. Calretinin was negatively correlated with the FS cell index in the autism and bipolar studies.
