# Supplementary material for: Measuring the Maturity of the Fast-Spiking Interneuron Transcriptional Program in Autism, Schizophrenia, and Bipolar Disorder
Source: PLoS One. 2012 Aug 24;7(8):e41215. doi: 10.1371/journal.pone.0041215 (PMC3427326; doi:10.1371/journal.pone.0041215)
Supplement: Table S4 — Multiple regression models with interneuron markers. Multiple linear regression modeling was performed with the FS cell index as the dependent variable. As CCK and VIP were also significantly correlated with the FS cell index in the autism, schizophrenia, and bipolar disorder studies, they were entered as covariates into multiple regression models. In all cases, the relationship between the FS cell index and parvalbumin levels remained significant. (PDF) [file pone.0041215.s004.pdf]

Study: Autism cortex

Index	Coeff	Std Err	p-value
PVALB	0.011	0.002	<0.0001***
CCK	0.006	0.002	0.0085**
VIP	0.004	0.001	0.0157*

Study: Schizophrenia dorsolateral prefrontal cortex

Index	Coeff	Std Err	p-value
PVALB	0.001	0.0004	0.0185*
CCK	0.006	0.001	0.0004***
VIP	0.001	0.001	0.4880

Study: Bipolar dorsolateral prefrontal cortex

Index	Coeff	Std Err	p-value
PVALB	0.010	0.002	0.0002***
CCK	0.004	0.003	0.2644
VIP	0.025	0.009	0.0049**

PVALB = parvalbumin
CCK = cholecystokinin
VIP = vasoactive intestinal peptide

Supplemental Table S4: Multiple regression models with interneuron markers. Multiple linear regression modeling was performed with the FS cell index as the dependent variable. As CCK and VIP were also significantly correlated with the FS cell index in the autism, schizophrenia, and bipolar disorder studies, they were entered as covariates into multiple regression models. In all cases, the relationship between the FS cell index and parvalbumin levels remained significant.
