# Supplementary material for: Measuring the Maturity of the Fast-Spiking Interneuron Transcriptional Program in Autism, Schizophrenia, and Bipolar Disorder
Source: PLoS One. 2012 Aug 24;7(8):e41215. doi: 10.1371/journal.pone.0041215 (PMC3427326; doi:10.1371/journal.pone.0041215)
Supplement: Table S5 — Chi-square analysis. For sub-tables 1–3, all genes were divided into 4 groups (up during FS cell development/up in disease; up during FS cell development/down in disease; down during FS cell development/up in disease; down during FS cell development/down in disease). Values were entered into an online chisquare 2×2 contingency table that used the Fisher's exact test for calculation of pvalues. In all cases, genes expression levels in the presence of disease changed in the opposite direction from that occurring during FS cell development (p<0.0001). For subtables 4–6, only genes that were significantly change in disease and during development were used. In all cases results remained highly significant (p<0.0001). (PDF) [file pone.0041215.s005.pdf]

	Up ASD	Down ASD
Up FS cell development	438(20.6%)	574(27.0%)
Down FS cell development	620(29.2%)	491(23.1%)

	Up SCZ	Down SCZ
Up FS cell development	459(21.6%)	597(28.1%)
Down FS cell development	599(28.2%)	468(22.1%)

	Up BPD	Down BPD
Up FS cell development	500(23.5%)	623(29.4%)
Down FS cell development	558(26.3%)	442(20.8%)

	Sig up ASD	Sig down ASD
Sig up FS cell development	51(14.0%)	104(28.6%)
Sig down FS cell development	151(41.5%)	58(15.9%)

	Sig up SCZ	Sig down SCZ
Sig up FS cell development	31(14.0%)	59(26.6%)
Sig down FS cell development	94(42.3%)	38(17.1)

	Sig up BPD	Sig down BPD
Sig up FS cell development	50(18.4%)	73(26.8%)
Sig down FS cell development	105(38.6%)	44(16.2%)

Supplemental table S5. Chi-square analysis. For sub-tables 1-3, all genes were divided into 4 groups (up during FS cell development/up in disease; up during FS cell development/down in disease; down during FS cell development/up in disease; down during FS cell development/down in disease). Values were entered into an online chi-square 2x2 contingency table that used the Fisher's exact test for calculation of p-values. In all cases, genes expression levels in the presence of disease changed in the opposite direction from that occurring during FS cell development ($p<0.0001$). For sub-tables 4-6, only genes that were significantly change in disease and during development were used. In all cases results remained highly significant ($p<0.0001$).
