# Supplementary material for: Measuring the Maturity of the Fast-Spiking Interneuron Transcriptional Program in Autism, Schizophrenia, and Bipolar Disorder
Source: PLoS One. 2012 Aug 24;7(8):e41215. doi: 10.1371/journal.pone.0041215 (PMC3427326; doi:10.1371/journal.pone.0041215)
Supplement: Table S6 — List of 95 genes that were significantly regulated in the same direction during disease and in an opposite direction during FS cell development. Genes are separated into 2 groups based on the direction of regulation. (PDF) [file pone.0041215.s006.pdf]

Upregulated during PV devlopment and down in DZ

Gene symbol	Gene name
asns	asparagine synthetase
atp5a1	ATP synthase, H+ transporting, mitochondrial F1 complex, alpha subunit, isoform 1
atp6v1a	ATPase, H+ transporting, lysosomal V1 subunit A
atp6v1b2	ATPase, H+ transporting, lysosomal V1 subunit B2
atp6v1c1	ATPase, H+ transporting, lysosomal V1 subunit C1
atp6v1d	ATPase, H+ transporting, lysosomal V1 subunit D
cab39	calcium binding protein 39
cckbr	cholecystokinin B receptor
chgb	chromogranin B
cse1l	chromosome segregation 1-like (S. cerevisiae)
cul3	cullin 3
dctn3	dynactin 3
ddx1	DEAD (Asp-Glu-Ala-Asp) box polypeptide 1
dlat	dihydrolipoamide S-acetyltransferase (E2 component of pyruvate dehydrogenase complex)
dld	dihydrolipoamide dehydrogenase
dlg3	discs, large homolog 3 (Drosophila)
dnm1l	dynamin 1-like
ebna1bp2	EBNA1 binding protein 2
eps15	epidermal growth factor receptor pathway substrate 15
gad1	glutamic acid decarboxylase 1
gad2	glutamic acid decarboxylase 2
grsf1	G-rich RNA sequence binding factor 1
iars	isoleucine-tRNA synthetase
idh3a	isocitrate dehydrogenase 3 (NAD+) alpha
idh3b	isocitrate dehydrogenase 3 (NAD+) beta
immt	inner membrane protein, mitochondrial
kcnab1	potassium voltage-gated channel, shaker-related subfamily, beta member 1
klhdc2	kelch domain containing 2
lrpprc	leucine-rich PPR-motif containing
map2k1	mitogen activated protein kinase kinase 1
mapk10	mitogen activated protein kinase 10
mapk9	mitogen activated protein kinase 9
mat2b	methionine adenosyltransferase II, beta
mdh1	malate dehydrogenase 1, NAD (soluble)
mtx2	metaxin 2
ndufs2	NADH dehydrogenase (ubiquinone) Fe-S protein 2
necap1	NECAP endocytosis associated 1
nnt	nicotinamide nucleotide transhydrogenase
npepps	aminopeptidase puromycin sensitive
nsf	N-ethylmaleimide sensitive fusion protein
pam	peptidylglycine alpha-amidating monooxygenase
pitpna	phosphatidylinositol transfer protein, alpha
prepl	prolyl endopeptidase-like
prps1	phosphoribosyl pyrophosphate synthetase 1
psmd1	proteasome (prosome, macropain) 26S subunit, non-ATPase, 1
psme3	proteaseome (prosome, macropain) 28 subunit, 3
ptpre	protein tyrosine phosphatase, receptor type, E
pvalb	parvalbumin
ralb	v-ral simian leukemia viral oncogene homolog B (ras related)
rgs4	regulator of G-protein signaling 4
rnf14	ring finger protein 14
rraga	Ras-related GTP binding A
serpini1	serine (or cysteine) peptidase inhibitor, clade I, member 1
sh3gl2	SH3-domain GRB2-like 2
smyd2	SET and MYND domain containing 2
ube2n	ubiquitin-conjugating enzyme E2N
ugp2	UDP-glucose pyrophosphorylase 2

uqcrc2	ubiquinol cytochrome c reductase core protein 2
vdac1	voltage-dependent anion channel 1
vsnl1	visinin-like 1

Downregulated during PV devlopment and up in DZ

**Gene symbol**	**Gene name**
acaa2	acetyl-Coenzyme A acyltransferase 2 (mitochondrial 3-oxoacyl-Coenzyme A thiolase)
ahnak	AHNAK nucleoprotein (desmoyokin)
aldh2	aldehyde dehydrogenase 2, mitochondrial
angptl4	angiopoietin-like 4
apoe	apolipoprotein E
bgn	biglycan
cdkn1a	cyclin-dependent kinase inhibitor 1A (P21)
csk	c-src tyrosine kinase
ddah2	dimethylarginine dimethylaminohydrolase 2
ddr1	discoidin domain receptor family, member 1
dpysl3	dihydropyrimidinase-like 3
eef1d	eukaryotic translation elongation factor 1 delta (guanine nucleotide exchange protein)
ephx1	epoxide hydrolase 1, microsomal
fxyd1	FXYD domain-containing ion transport regulator 1
fyn	Fyn proto-oncogene
gnb2l1	Guanine nucleotide binding protein (G protein), beta polypeptide 2 like 1
h2afj	H2A histone family, member J
marcksl1	MARCKS-like 1
ndrg2	N-myc downstream regulated gene 2
necap2	NECAP endocytosis associated 2
nkiras2	NFKB inhibitor interacting Ras-like protein 2
nme4	expressed in non-metastatic cells 4, protein
ntsr2	neurotensin receptor 2
pax6	paired box gene 6
ptn	pleiotrophin
ptpn13	protein tyrosine phosphatase, non-receptor type 13
rab31	RAB31, member RAS oncogene family
ramp1	receptor (calcitonin) activity modifying protein 1
rhoc	ras homolog gene family, member C
rps19	ribosomal protein S19
sec61a1	Sec61 alpha 1 subunit (S. cerevisiae)
slc3a2	solute carrier family 3 (activators of dibasic and neutral amino acid transport), member 2
tnfrsf1a	tumor necrosis factor receptor superfamily, member 1a
txnip	thioredoxin interacting protein
ung	uracil DNA glycosylase

Supplemental table s6. List of 95 genes that were significantly regulated in the same direction during disease and in an opposite direction during FS cell development. Genes are separated into 2 groups based on the direction of regulation.
