# Supplementary material for: Measuring the Maturity of the Fast-Spiking Interneuron Transcriptional Program in Autism, Schizophrenia, and Bipolar Disorder
Source: PLoS One. 2012 Aug 24;7(8):e41215. doi: 10.1371/journal.pone.0041215 (PMC3427326; doi:10.1371/journal.pone.0041215)
Supplement: Table S7 — Top chart is pathway analysis using DAVID of the 60 genes that were upregulated during FS cell development and downregulated in autism, bipolar disorder, and schizophrenia. Middle chart is pathway analysis using DAVID of 85 genes downregulated in autism, bipolar, and schizophrenia. Bottom chart is pathway analysis using DAVID of the 1023 genes that were upregulated in FS cells over development. Pathways enriched in all are starred. (PDF) [file pone.0041215.s007.pdf]

Upregulated in FS cells AND downregulated in disease

Category	Term	count	%	PValue	Fold Enrich	Benjamini
GOTERM_BP_FAT	*generation of precursor metabolites and energy	12	20	1.84E-06	5.92	0.0008
GOTERM_BP_FAT	proton transport	6	10	1.53E-05	16.29	0.0032
GOTERM_BP_FAT	hydrogen transport	6	10	1.53E-05	16.29	0.0032
GOTERM_CC_FAT	proton-transporting two-sector ATPase complex, c	5	8.33	4.00E-05	21.06	0.0051
GOTERM_BP_FAT	oxidative phosphorylation	6	10	8.07E-05	12	0.0113
GOTERM_BP_FAT	ATP synthesis coupled proton transport	5	8.33	1.64E-04	15.83	0.0172
GOTERM_BP_FAT	energy coupled proton transport	5	8.33	1.64E-04	15.83	0.0172
GOTERM_BP_FAT	*monovalent inorganic cation transport	8	13.3	2.04E-04	6.08	0.0171
GOTERM_BP_FAT	ion transmembrane transport	5	8.33	2.33E-04	14.62	0.0162
SP_PIR_KEYWORDS	Hydrogen ion transport	5	8.33	2.42E-04	14.48	0.034
KEGG_PATHWAY	*Oxidative phosphorylation	7	11.7	3.72E-04	6.51	0.0282
GOTERM_BP_FAT	ribonucleotide biosynthetic process	6	10	5.73E-04	8.14	0.034
GOTERM_CC_FAT	*mitochondrion	18	30	7.54E-04	2.25	0.0314

Downregulated in disease

Category	Term	Count	%	PValue	Fold Enrichn	Benjamini
GOTERM_BP_FAT	*generation of precursor metabolites and energy	12	14.1	8.48E-05	4.09	0.048733
GOTERM_BP_FAT	proton transport	6	7.06	9.86E-05	11.26	0.028607
GOTERM_BP_FAT	hydrogen transport	6	7.06	9.86E-05	11.26	0.028607
GOTERM_CC_FAT	proton-transporting two-sector ATPase complex, c	5	5.88	1.64E-04	14.90	0.026806
GOTERM_BP_FAT	oxidative phosphorylation	6	7.06	4.94E-04	8.29	0.092524
GOTERM_BP_FAT	ATP synthesis coupled proton transport	5	5.88	7.14E-04	10.94	0.099864
GOTERM_BP_FAT	energy coupled proton transport, down electroche	5	5.88	7.14E-04	10.94	0.099864
SP_PIR_KEYWORDS	Hydrogen ion transport	5	5.88	9.48E-04	10.22	0.151364
GOTERM_BP_FAT	ion transmembrane transport	5	5.88	1.00E-03	10.10	0.111459
GOTERM_CC_FAT	proton-transporting two-sector ATPase complex	5	5.88	1.02E-03	9.94	0.081277
KEGG_PATHWAY	*Oxidative phosphorylation	7	8.24	1.44E-03	5.15	0.121341
GOTERM_MF_FAT	proton-transporting ATPase activity, rotational mec	4	4.71	2.10E-03	13.64	0.375094
GOTERM_BP_FAT	*monovalent inorganic cation transport	8	9.41	2.11E-03	4.20	0.187242
GOTERM_MF_FAT	cofactor binding	8	9.41	2.88E-03	3.98	0.275862
GOTERM_BP_FAT	ribonucleotide biosynthetic process	6	7.06	3.21E-03	5.63	0.237204
GOTERM_CC_FAT	*mitochondrion	21	24.7	3.95E-03	1.86	0.196724

Upregulated in FS cells

Category	Term	Count	%	PValue	Fold Enrich	Benjamini
GOTERM_CC_FAT	*mitochondrion	171	16.7	9.73E-08	1.31	0.0000
SP_PIR_KEYWORDS	mitochondrion	112	11	1.57E-06	1.37	0.0008
SP_PIR_KEYWORDS	transport	171	16.7	7.57E-05	1.22	0.0180
GOTERM_CC_FAT	mitochondrial part	84	8.22	1.96E-04	1.33	0.0459
GOTERM_BP_FAT	cofactor metabolic process	34	3.33	2.42E-04	1.61	0.5147
KEGG_PATHWAY	*Oxidative phosphorylation	26	2.54	3.47E-04	1.70	0.0609
GOTERM_BP_FAT	*generation of precursor metabolites and energy	52	5.09	3.66E-04	1.44	0.4208
GOTERM_BP_FAT	cation transport	57	5.58	3.69E-04	1.41	0.3069
UP_SEQ_FEATURE	Mitochondrion	69	6.75	3.71E-04	1.36	0.6305
SP_PIR_KEYWORDS	transit peptide	69	6.75	6.30E-04	1.34	0.0962
GOTERM_BP_FAT	*monovalent inorganic cation transport	36	3.52	6.81E-04	1.53	0.3983

Supplemental table s7. Top chart is pathway analysis using DAVID of the 60 genes that were upregulated during FS cell development and downregulated in autism, bipolar disorder, and schizophrenia. Middle chart is pathway analysis using DAVID of 85 genes downregulated in autism, bipolar, and schizophrenia. Bottom chart is pathway analysis using DAVID of the 1023 genes that were upregulated in FS cells over development. Pathways enriched in all are starred.
